# Long-term impact of disclosing amyloid PET results to individuals with subjective cognitive decline

**DOI:** 10.1186/s13195-026-02026-3

**Published:** 2026-03-27

**Authors:** Jetske van der Schaar, Wiesje M. van der Flier, Leonie N. C. Visser, Elsmarieke van de Giessen, Argonde C. van Harten, Sietske A. M. Sikkes, Mardou S. S. A. van Leeuwenstijn-Koopman, Heleen M. A. Hendriksen, Calvin Trieu, Annelien L. Bredenoord, Mariette A. van den Hoven, Eva C. A. Asscher

**Affiliations:** 1 Section Genomics of Neurodegenerative Diseases and Aging, Department of Human Genetics, Amsterdam UMC location VUmc, De Boelelaan 1117, Amsterdam, 1081 HV Netherlands; 2 Alzheimer Center Amsterdam, Neurology, Vrije Universiteit Amsterdam, Amsterdam UMC location VUmc, De Boelelaan 1117, Amsterdam, 1081 HV The Netherlands; 3 Amsterdam Neuroscience, Neurodegeneration, Research & Diagnostics Center (RDC) - ADORE, Van der Boechorststraat 6B, Amsterdam, 1081 BT The Netherlands; 4 Alzheimer Nederland, Stationsplein 121, Amersfoort, 3818 LE The Netherlands; 5 Department of Epidemiology & Data sciences, Vrije Universiteit Amsterdam, Amsterdam UMC location VUmc, De Boelelaan 1117, Amsterdam, 1081 HV The Netherlands; 6 Amsterdam Public Health, Quality of Care, Van der Boechorststraat 7, Amsterdam, 1081 BT The Netherlands; 7 Department of Medical Psychology, Amsterdam UMC, location AMC, Meibergdreef 9, Amsterdam, 1105 AZ The Netherlands; 8 Department of Bioethics and Health Humanities, Julius Center for Health Sciences and Primary Care, University Medical Center Utrecht, Utrecht University, Universiteitsweg 100, Utrecht, 3584 CG The Netherlands; 9 Department of Radiology & Nuclear Medicine, Vrije Universiteit Amsterdam, Amsterdam UMC location VUmc, De Boelelaan 1117, Amsterdam, 1081 HV The Netherlands; 10 Amsterdam Neuroscience, Brain Imaging, Research & Diagnostics Center (RDC) - ADORE, Van der Boechorststraat 6B, Amsterdam, 1081 BT The Netherlands; 11 Faculty of Behavioural and Movement Sciences, Clinical, Neuro and Developmental Psychology, Vrije Universiteit Amsterdam, De Boelelaan 1105, Amsterdam, 1081 HV The Netherlands; 12 Department of Clinical Chemistry, Neurochemistry Lab and Biobank, Amsterdam Neuroscience, Amsterdam UMC location VUmc, De Boelelaan 1117, Amsterdam, 1081 HV The Netherlands; 13 Erasmus School of Philosophy, Erasmus University Rotterdam, Burgemeester Oudlaan 50, Rotterdam, 3062 PA The Netherlands; 14 Department of Ethics, Law and Humanities, Amsterdam UMC, De Boelelaan 1089a, Amsterdam, The Netherlands

**Keywords:** Alzheimer’s disease, Patient perspective, Patient experience, Subjective cognitive decline, Biomarker disclosure, Amyloid pet, Psychosocial impact, Behavioral impact, Physician-assisted suicide, Euthanasia

## Abstract

**Background:**

Biomarker assessments increasingly inform the diagnostic evaluation and treatment decisions in Alzheimer disease (AD). However, evidence on the impact of amyloid positron emission tomography (PET) disclosure is primarily derived from studies of cognitively unimpaired trial participants with follow-up limited to 18 months. In contrast, long-term implications for individuals with cognitive concerns who seek medical evaluation in memory clinics remain unknown. We aimed to examine the psychosocial and behavioral impact three years after amyloid PET disclosure in individuals presenting with subjective cognitive decline (SCD) at a memory clinic.

**Methods:**

In-depth semi-structured interviews were conducted with 17 participants from the Subjective Cognitive Impairment Cohort (SCIENCe) (67 ± 7 years; 5 female; 10 amyloid positive) 35 ± 4 months post-disclosure. All had SCD at imaging; one had progressed to mild cognitive impairment (MCI) at interview. Verbatim transcripts were analyzed inductively.

**Results:**

Participants’ motivations for testing and long-term adaptation to the results were strongly shaped by cognitive concerns and personal experiences of dementia in relatives. All demonstrated accurate comprehension of their amyloid status. Those with negative scans described immediate relief and reattributed memory lapses to normal aging, while recognizing that the reassurance was provisional. Although positive scans provoked initial shock and fear of deterioration, participants valued information over uncertainty, and over time, fear attenuated as they perceived no rapid decline. Both groups regarded results as meaningful and personally actionable, informing health behavior, life priorities, and preparations for future decline. Fourteen of 17 participants spontaneously mentioned considering options for self-determined end-of-life. Regardless of amyloid status, most participants shared their result with close relatives and friends, some also informed colleagues or acquaintances, while others limited communication to avoid stigma, protect loved ones, or reduce the burden of repeated explanations. Participants valued testing, expressed no regret, and would choose disclosure again.

**Conclusions:**

Three years after disclosure, participants generally had adjusted to living with their imaging result, finding personal meaning and practical engagement without reporting ongoing psychological harm. Some reported residual concerns and uncertainty, irrespective of amyloid status. These findings offer timely guidance for clinicians and patients as biomarker disclosure is more widely incorporated into routine practice.

**Supplementary Information:**

The online version contains supplementary material available at 10.1186/s13195-026-02026-3.

## Background

Amyloid positron emission tomography (PET) imaging enables in vivo detection of β-amyloid pathology up to decades before the clinical manifestation of Alzheimer’s disease (AD) [1–3]. This technological advance has created an opportunity to study the disease in its earliest biological stages, aiming to improve understanding of its progression and to evaluate interventions that may delay or prevent symptom onset [4, 5]. Consequently, clinical trials increasingly recruit cognitively healthy individuals with β-amyloid pathology, as longitudinal studies have shown that this population is at elevated risk of cognitive decline [6, 7].

The majority of participants express interest in learning their amyloid PET status [8–10]. While this may offer biological information, it also raises ethical, psychological, and societal concerns about the potential consequences of returning biomarker results [11]. In response, a growing body of empirical research has examined the effects of disclosure to individuals who do not meet diagnostic criteria for dementia, generally showing no substantial psychological harm [12]. However, our systematic review found these studies were predominantly conducted in trial settings with cognitively unimpaired participants [12–20]. Moreover, follow-up times were limited to 18 months.

Less is known about the impact of disclosure in persons who present at a memory clinic with subjective cognitive decline (SCD), defined by self-reported cognitive complaints with neuropsychological test performance within normal limits [21]. This gap is clinically relevant, as these individuals present to memory clinics seeking medical evaluation for their concerns and represent the next clinical population in whom amyloid biomarker disclosure is likely to be implemented. Compared to cognitively unimpaired trial participants, those with SCD and abnormal AD biomarkers face a higher risk of subsequent cognitive decline [22–24]. Therefore, understanding how they respond to and live with knowledge of their amyloid status in the longer term is essential to guide responsible implementation of biomarker disclosure in clinical practice.

We previously disclosed amyloid PET results to 57 individuals with SCD within a structured research setting, evaluating their experiences and outcomes over a six-month follow-up period. In that study, 57 participants (37% women; mean age 66 ± 8 years) received their amyloid PET result. They valued the disclosure process regardless of amyloid status and reported low levels of emotional distress and regret [18]. In addition, a wide range of needs was expressed, reflecting a desire not only for information and clarification but also personalized care and support [25]. Building on this earlier work, we conducted a follow-up interview study approximately three years after disclosure. Our aim was to gain in-depth insight into how individuals with SCD reflect on their decision to learn their amyloid PET status in the long term, and how they experience the psychological, social, and behavioral implications of living with this knowledge.

## Methods

### Study setting

This observational study was embedded in the Subjective Cognitive Impairment Cohort (SCIENCe) [26] at Alzheimer Center Amsterdam, Amsterdam UMC. It includes individuals who were referred to our memory clinic by their general practitioner, and a neurologist or geriatrician in the case of a second opinion for evaluation of cognitive complaints. They underwent a standardized diagnostic workup comprising neurological, physical, and neuropsychological assessments, routine laboratory testing, as well as brain magnetic resonance imaging (MRI) [27, 28]. In a multidisciplinary consensus meeting, they were classified as having SCD when cognitive performance was within normal limits, and criteria for mild cognitive impairment (MCI), dementia, or other neurological or psychiatric disorders (e.g., major depression, schizophrenia) were not met. Those with SCD who consent to participate in the SCIENCe study are invited for annual follow-up visits that consisting of clinical assessment, neuropsychological testing, and diagnostic re-evaluation.

### Amyloid PET disclosure

A subset of SCIENCe participants underwent an amyloid PET scan (procedural details [24]), which was visually rated as “normal” or “abnormal” by an experienced nuclear medicine physician. Those who had opted to learn their status were informed by a neurologist, who explained the results as showing either absence or presence of abnormal amyloid protein in the brain, indicating a low five-year (negative) or elevated 20-year risk (positive) of developing dementia due to AD (disclosure process [18]).

### Semi-structured interviews

For the current study, JS recruited SCIENCe participants (by phone and email) who had learned their amyloid PET result in 2020–2021, and remained cognitively able to provide informed consent. During the follow-up period, no additional AD biomarker results were disclosed to them. Purposive sampling was used by approaching candidates before their annual follow-up visits, and aiming for overrepresentation of individuals with abnormal amyloid PET results. JS (a female PhD candidate trained in qualitative methods) conducted one-time in-depth, semi-structured interviews between January and July 2024, each lasting approximately 45 min and taking place at the memory clinic, at participants’ homes, or by phone, according to their preference. In one case a partner was present as well. Field notes were made immediately afterwards. Recruitment continued until data saturation was achieved, indicating that no new concepts emerged from successive interviews. We used a topic guide that covered reflections on the decision to learn their amyloid status, the considerations involved at the time, and how they viewed this choice in retrospect, as well as the psychological, social, and behavioral consequences of living with this knowledge, including potential changes in life priorities and plans (*Supplement 1*). Results are reported in accordance with the Consolidated Criteria for Reporting Qualitative Research (COREQ) guidelines [29].

### Qualitative analysis

Audio recordings were transcribed verbatim, and the resulting pseudonymized transcripts were managed using MAXQDA (version 22.1.1; VERBI Software). Two authors (JS and EA) performed an inductive thematic analysis [30], identifying concepts, which were iteratively refined and organized into (sub)categories and overarching themes. To ensure consistency and accuracy, five transcripts were double-coded, and discrepancies were discussed until consensus was reached. The codebook was further developed over the course of the analysis, and once a stable coding framework had been agreed, JS applied it to all transcripts, revisiting earlier interviews where needed. Verbatim quotes were minimally edited for clarity and readability; expletives were retained to preserve participants’ original expression.

## Results

### Participants’ characteristics

We approached twenty candidates, of whom two declined participation, one could not be scheduled, resulting in 17 completed interviews. Participants had learned their amyloid PET status on average 35 ± 4 months earlier. At the time, 10/17 (59%) tested amyloid positive (A+) and seven (41%) negative (A-). Demographic and clinical characteristics at the time of amyloid PET status disclosure and interview are presented in Table 1. The sample included five female participants (5/17, 29%), the mean age when interviewed was 67 ± 7 years old, and educational attainment was generally high [31].

**Table 1:** Demographic and clinical characteristics of participants at the time of amyloid PET status disclosure and the follow-up interview, stratified by group (negative / positive)

	all (*n* = 17)	negative (*n* = 7)	positive (*n* = 10)	*p*-value
Sex (*n* (%) female)	5 (29%)	3 (43%)	2 (20%)	0.59
Education (mean ± sd)	6 ± 1	6 ± 1	6 ± 1	1.00
Marital status				0.12
Married	10 (59%)	6 (86%)	4 (40%)	
Cohabitating	1 (6%)		1 (10%)	
Unmarried	2 (12%)		2 (20%)	
Widowed	1 (6%)	1 (14%)		
Missing	3 (18%)		3 (30%)	
At time of amyloid PET disclosure				
Age (mean ± sd years)	64 ± 8	64 ± 7	65 ± 9	0.81
MMSE (mean ± sd)	29 ± 1	29 ± 1	29 ± 1	0.93
Diagnosis				1.00
SCD (*n* (%))	17 (100%)	7 (100%)	10 (100%)	
At time of follow-up interview				
Age (mean ± sd years)	67 ± 7	67 ± 7	67 ± 8	0.92
MMSE (mean ± sd)	29 ± 1	29 ± 1	29 ± 1	0.56
Diagnosis				1.00
SCD (*n* (%))	16 (94%)	7 (100%)	9 (90%)	
MCI (*n* (%))	1 (6%)	0 (0%)	1 (10%)	

Education was rated using the Dutch Verhage system (range: 1-7), with 1 being the lowest (less than primary school) and 7 being the highest (academic degree) [31]

Differences between amyloid-positive and -negative individuals were analyzed using one-way ANOVA, Mann-Whitney U, or Fisher’s Exact Test when appropriate

*MCI* Mild Cognitive Impairment, *MMSE* Mini-Mental State Examination, *PET* Positron Emission Tomography, *SCD* Subjective Cognitive Decline

Illustrative quotes corresponding to the theme and subthemes are provided in *Supplement 2*.

### Motivations for learning amyloid PET status

When asked about their motivation for learning their amyloid PET scan results, most participants cited a family history of dementia. Many described witnessing cognitive decline in loved ones as a harrowing experience that profoundly affected them: “[I]t was an absolutely hor-ri-ble process. […] Our sweet, gentle mother became suspicious, angry, and fearful. […] And I was, I am, very afraid that I will get it too.” (P04, A-). Consequently, they expressed a strong desire to gain insight into their brain health “to know what’s going on, and where it’s headed” (P01, A+). This attitude was often rooted in character or principle: “I’m not the kind of person who buries their head in the sand. […] If tests are being done, whether the result is negative or positive, I do want to know.” (P08, A-). Some respondents sought explanations for their perceived cognitive decline as it unsettled them: “It made me feel insecure, like, I don’t remember things, is something wrong with me?” (P10, A+), and they hoped to find out if anything could be done. Notably, half of the participants reported perceiving no drawbacks to learning their amyloid PET results: “Why wouldn’t I want to know?” (P18, A+). In contrast, others, however, mentioned drawbacks, including emotional impact, uncertainty about their prognosis, and disappointment about a lack of clinical utility.

### Comprehension of amyloid PET status

All participants showed accurate comprehension of their amyloid PET status, although one did not specifically recall their test result and correctly reconstructed it instead: “If I don’t remember it, then there was nothing special going on” (P07, A-). Others described a negative scan as “good”, defining it as the absence of amyloid, proteins or plaques in the brain, which they explained as “no physically detectable features of the process” (P09, A-), concluding: “Alzheimer’s isn’t there either” (P08, A-). They understood this as “a snapshot”, indicating they would not “be affected for the next ten years” (P09, A-), while acknowledging “it wasn’t a 100% guarantee” they would never develop the disease (P06, A-). Conversely, an abnormal PET scan result was referred to as “bad news”. Respondents reported having increased levels of amyloid, proteins or plaques, which they considered “not something you want in your head” (P01, A+), and took to mean “you basically have the early stage of Alzheimer’s” (P13, A+). While this was characterized as being at high risk or “almost certainly” progressing to dementia, they emphasized that “it can take *years*” (P17, A+) and “it’s not set in stone that you’ll develop it” (P16, A+).

### Emotional impact of amyloid PET result

Participants described strong and evolving emotional responses to receiving their amyloid PET status. A favorable result prompted immediate happiness and relief, intensified by anticipatory stress and recognition that the outcome could have been worse. For many, it provided “peace of mind” by reducing concerns about developing dementia: “these are the facts for me now, I trust them, this becomes my starting point” (P05, A-). Many remained aware of cognitive decline, with several reattributing memory lapses to normal aging: “it is not that bad compared to people my age” (P06, A-). The majority recognized the result as provisional rather than definitive, describing it as a “snapshot” offering respite: “seize the day, things will be fine for a while” (P11, A-). In contrast, learning about elevated amyloid PET levels was mostly experienced as shock and disappointment, accompanied by the dread of what it might imply: “like, you’re thinking: whoa, here we go” (P12, A+), and the realization that “nothing could be done about it” (P13, A+). Several participants valued clarity over “constantly asking yourself: Jesus, why do I keep forgetting all these things all the time?” (P02, A+). Others highlighted that amyloid pathology was detected, dementia had not developed (yet). Although most noticed gradual decline, distress often attenuated over the following years when they perceived no rapid deterioration: “as time goes by and I do not see a disastrous decline, that fear fades, or at least becomes less” (P02, A+). Across groups, participants continued to monitor their memory, with some explicitly questioning at what point forgetfulness was still part of normal ageing or might begin to reflect early AD.

### Actionability of amyloid PET status

Learning their amyloid PET result prompted participants to reflect on their remaining life span, regardless of their status: “Assuming you only have 15, 10, or 5 conscious years left - though you never know - what do you want then and what don’t you want?” (P05, A-). Those testing positive described shifting priorities toward living in the moment, spending time with family, going on holidays, and seizing opportunities as they arise. Moreover, their perceived cognitive decline or the presence of amyloid pathology served as “a motivator to maintain a good and healthy lifestyle” (P10, A+), stimulating some to make substantial changes to keep their “mind in good shape”, while other respondents saw little room or motivation for improvement. Many participants anticipated that enrolling in research would ultimately lead to “a moment when [the disease] can be halted” (P03, A+), with some also hoping to benefit personally through early access to clinical trials or novel treatments, although one participant reported disappointment due to limited opportunities. Regardless of clinical utility or their biomarker status, participants emphasized the importance of preparing for cognitive decline across multiple domains: “your life plans, to your housing, to, well, your entire social context, actually.” (P06, A-). Remarkably, the vast majority (independent of amyloid status) spontaneously and unprompted mentioned considering or exploring possibilities for euthanasia, physician-assisted dying, and/or suicide as contingent options if they were to reach an advanced stage of dementia, rather than as current intent. These reflections often related to anticipated loss of autonomy, fear of burdening relatives, and uncertainty about determining the ‘right time’ while still competent: “I don’t want to go through the entire course of the disease. I want […] to stop it early. Life like that has no value for me. And I don’t want my children and my husband to remember me that way.” (P04, A-).

### Sharing amyloid PET status with others

Most participants discussed their amyloid PET result with their inner circle of immediate family and close friends. Irrespective of their test result, some also informed colleagues, and acquaintances from community roles or leisure groups. This openness reflected their “nature to share” experiences with others (P08, A-), their belief that loved ones should be aware of “things that are important for them too” (P14, A+ ), or their wish to prevent misunderstandings: “if I start having trouble remembering the game, well, then they’ll also know the cause” (P16, A+). Other respondents were more cautious in sharing what they considered to be “sensitive information” (P03, A+). They sought to limit the emotional toll of “explaining to everyone and their dog what it means to get news like this” (P07, A-), avoid stigma or discrimination, noting, “if your employer finds out, then it’s pretty much the end of your career” (P05, A-), or protect their loved ones: ‘I don’t think I should burden [my wife] with that.” (P18, A+).

### Perceived significance of amyloid PET testing

Finally, participants reflected on the longer-term impact of having known their amyloid PET status for several years. For some, the personal significance of this knowledge had shifted over time. One respondent described a greater awareness of mortality, knowing “it’s my turn, and sooner than normal”, which prompted a sense of urgency (P02, A+), while another came to see that life with cognitive decline “is still worth living” and realized: “I don’t want to die yet.” (P13, A+). Participants reported no regret, and despite any emotional challenges, all would choose to undergo biomarker testing again. Some stated they would have “preferred to do it even earlier” (P10, A+), emphasized that although “it’s bad news, I still want to know” (P13, A+), or highlighted that there is no downside to “trying to do the things you want to do now” (P16, A+). When asked what they would advise others in a similar situation, they indicated that “it really depends on people’s attitudes” ( 11, A-), explaining that not everyone might “want to face that reality so directly” (P06, A-), while one recommended repeated measurements “if you want to know what the prognosis is.” (P18, A+).

## Discussion

Our main findings are that among individuals presenting at a memory clinic with subjective symptoms, formative encounters with dementia in loved ones strongly influenced their perspectives on getting tested and experiences of learning the result. After three years, acute emotional responses to amyloid PET disclosure had generally shifted toward sustained adjustment, with reassurance tempered by the provisional nature of the scan, and distress attenuating in the absence of rapid decline. Both amyloid-positive and amyloid-negative participants regarded the information as personally actionable, guiding health behavior, future planning, and end-of-life considerations. Ultimately, participants reported no regret and would choose testing again.

Whereas previous work on biomarker disclosure has largely involved participants in prevention trials [12, 32–34], , we included patients with SCD who presented at a memory clinic for evaluation of perceived symptoms. Earlier studies in research populations with cognitively unimpaired individuals have reported a wide variety of reasons for pursuing biomarker information, including a desire to gain insight into brain health, to learn about risk of dementia, and to confirm or dispel memory concerns [12, 33, 34]. Some further indicate that interest in testing was more common among individuals with a family history and those who perceived themselves at higher risk of developing the disease, and to a lesser extent among those reporting subjective memory problems [8, 35, 36]. In our cohort, motivation for learning their biomarker status and longer-term adjustment to its result were often shaped by the profound impact of losing loved ones to dementia, which instilled a lasting fear of developing the disease themselves. When confronted with their own emerging cognitive concerns, participants sought to learn whether amyloid pathology was present and, if so, to prepare for future decline, including consideration of self-determined end-of-life decisions. These observations suggest that witnessing the disease in close relatives and perceiving symptoms in oneself can amplify the significance of biomarker disclosure, underscoring the need for personalized counseling and tailored support in clinical contexts. Accordingly, shared decision-making between patient and clinician should encompass personal values and circumstances, as well as the test’s limitations and the implications, to determine whether testing is to be pursued.

While disclosure initially elicited strong reactions, from relief after negative scans to shock after positive scans, subsequent accounts emphasized adaptation, with reassurance often recognized as provisional and distress frequently diminishing over time. Earlier work within the same memory-clinic cohort showed that disclosure was well tolerated at six months, with no increase in anxiety or negative affect regardless of amyloid status [18], consistent with studies in cognitively unimpaired individuals showing that any psychological effects typically resolve within months [12, 15, 16, 19, 20]. Our data add a longer-term perspective, showing that emotional responses continue to evolve over several years as individuals re-interpret the result through lived experience. For clinical implementation, this underscores the relevance of offering a planned follow-up contact after disclosure, to revisit the implications of the result, address emerging concerns, and offer additional support when indicated.

Participants regarded amyloid disclosure as actionable. In the absence of access to disease-modifying treatment and with only limited opportunities for trial participation, the perceived utility of this information for individuals with SCD was largely personal rather than clinical. Accounts described reconsidering priorities, preparing for decline, and in some cases making lifestyle changes, which is in line with previous reports that individuals may use biomarker information to adapt their plans and behavior [12, 17, 32, 33]. At the same time, interviewees understood that amyloid PET provides probabilistic rather than certain information about future dementia risk. These empirical observations contrasts with theoretical discussions that question whether limited prognostic certainty constrains the personal utility [11]. While ethical analyses have emphasized ambiguity and lack of clear actionability, our data show that participants interpreted and applied their results in concrete ways, using them to guide decisions about health and planning. Taken together, these findings highlight the importance of pre-test counseling to align expectations with current clinical reality. In particular, clinicians should address uncertainty about individual timing and pace of progression, as well as practical implications regarding trial eligibility and access to novel treatments.

Beyond these behavioral responses, we found that most participants spontaneously and unprompted contemplated self-determined end-of-life options. These reflections were generally framed as conditional, future-oriented planning in the event of advanced dementia, rather than as current suicidal intent. Earlier studies in similar populations noted such considerations but generally among a minority [36–39]; however, these investigations focused on individuals’ expectations in the context of hypothetical biomarker testing. In one interview study examining actual disclosure, these views were attributed to pre-existing attitudes toward physician-assisted death [40], an interpretation supported by a large trial showing no effect of amyloid status on suicidality [41]. A plausible explanation for the higher frequency of such reflections in our cohort, is the specific profile of this group: individuals with SCD who both perceived emerging cognitive problems and had witnessed dementia in close relatives, which may heighten concerns about future dependency and loss of autonomy. The legal and cultural context may also play a role, as euthanasia is regulated in the Netherlands and Belgium, and has occasionally been requested and granted in patients with dementia [42–44]. These considerations focused on a hypothetical future dementia stage and were voiced by both amyloid-positive and amyloid-negative participants, suggesting that such thoughts arise from broader concerns about future decline and may be part of advance care planning shaped by personal history and the surrounding legal and cultural context, rather than a harmful disclosure-induced response. In practice, clinicians and researchers should assess whether statements reflect future-oriented planning versus current suicidal intent and arrange additional support or referral when indicated.

Comprehension of amyloid imaging was consistently accurate irrespective of amyloid status. This aligns with prior research showing that cognitively unimpaired individuals generally understand their biomarker status [17, 33, 45, 46], and demonstrates that this knowledge can be maintained over a longer period, although the salience of negative findings may diminish with time. Participants described abnormal scans as serious yet uncertain, reflecting the unpredictability of prognosis, while normal pathology levels were regarded as reassuring but provisional, realizing that they could still become elevated. The durability and nuance of this understanding suggest that clear explanation during disclosure and follow-up can enable laypersons to distinguish between the pathology of AD and the syndrome of dementia, challenging concerns that such distinctions exceed public comprehension [47]. Together with earlier reports on this cohort, highlighting the value of follow-up opportunities for questions [18, 25], these observations underscore the importance of clear communication to support sustained understanding of biomarker results.

Participants differed in whether and with whom they shared their amyloid status. Most informed immediate family or trusted friends, while some extended this to colleagues or acquaintances. Openness was motivated by a wish to enable understanding and support or prevent misinterpretation of memory lapses. Others were more restrained, seeking to protect loved ones from distress or to avoid stigma and discrimination. These patterns echo earlier reports that many convey results to their closest relations, while concerns about negative social consequences can limit wider communication [17, 45, 48]. For clinical practice, this points to the need for counseling to help individuals consider with whom and how to share their status, and how potential consequences might unfold.

None of our participants expressed regret about learning their amyloid status, and all stated they would choose to do so again. This aligns with previous studies reporting high willingness to make the same decision if offered the same opportunity [15, 49]. When asked what advice they would give others, they emphasized the choice is deeply personal, recognizing not everyone may wish to confront such knowledge, and the response is equally individual. For some it brought a sharpened awareness of mortality and urgency, for others it sustained a sense of continuity and acceptance. Yet for all the impact evolved over time. These observations highlight that the psychosocial and behavioral consequences of disclosure are not fixed but unfold across years, underscoring the importance of counseling that recognizes both personal variation and long-term adaptation. Future studies should examine these trajectories in larger and more diverse SCD cohorts and across different healthcare and legal contexts.

### Strengths and limitations

A strength of this study is the three-year follow-up after amyloid disclosure, exceeding the periods of up to 18 months reported in earlier work, and allowing us to capture longer-term psychosocial and behavioral consequences. Focusing on individuals with SCD is clinically relevant, as they frequently present to memory clinics or brain health services, seeking clarity about their cognitive health. This population has been underrepresented in disclosure research, which has largely involved cognitively unimpaired trial participants, yet it is important to study them as symptomatic persons may experience greater emotional distress after disclosure than asymptomatic peers [14]. The qualitative design further provided the depth to examine participant’s lived experience in detail. In addition, the European setting adds breadth to a literature that has been dominated by US cohorts [12], particularly in relation to end-of-life considerations in a context where euthanasia is legally regulated with wider applicability, including in dementia.

At the same time, several limitations should be acknowledged. Our participants represent a highly selective subgroup: all had SCD, consented to amyloid PET imaging, chose to learn their status, and remained engaged for a three-year follow-up. This self-selection likely yielded a cohort that is motivated, health-literate, and psychologically resilient. Their generally high educational attainment may have further facilitated comprehension and adjustment, consistent with evidence linking education to greater capacity to interpret and cope with positive results [20]. Generalizability is limited by the sample’s demographic and contextual profile: participants were relatively young (mean age 67 years), predominantly men (12/17), White, and recruited from a Western European memory clinic within a specific healthcare and legal context. Because disclosure occurred within a research protocol after routine memory clinic evaluation, we cannot disentangle whether or how prior diagnostic feedback shaped participants’ responses to learning their amyloid PET result. Future studies should include larger, more diverse samples along these dimensions and examine whether experiences of disclosure and longer-term adaptation differ across clinical settings and social contexts. Despite these caveats, the findings nonetheless suggest that individuals who actively choose amyloid disclosure can understand, tolerate, and integrate this knowledge, supporting the feasibility of offering such choices in clinical practice.

## Conclusion

This study offers a rare view on the long-term impact of amyloid PET disclosure in individuals with SCD presenting at a memory clinic. After three years, participants understood and valued knowledge of their status, found it emotionally manageable and personally actionable, and expressed no regret. Their cognitive concerns, compounded by family experiences with dementia, shaped motivations for testing, living with the result, and reflections on mortality. They used the information to guide health behavior, re-evaluate life priorities, prepare for cognitive decline and consider options for a self-determined death. These findings offer timely guidance for clinicians and patients as biomarker disclosure becomes increasingly integrated into routine practice.

## Supplementary Information

Supplementary Material 1.

Supplementary Material 2.

Supplementary Material 3.

## Data Availability

The data that support the findings of this study are available from the corresponding author, upon reasonable request.

## Abbreviations

AD: Alzheimer’s disease
COREQ: Consolidated Criteria for Reporting Qualitative Research
MCI: Mild cognitive impairment
MRI: Magnetic resonance imaging
PET: Positron emission tomography
SCD: Subjective cognitive decline
SCIENCe: Subjective Cognitive Impairment Cohort
