# Supplementary material for: Long-term impact of disclosing amyloid PET results to individuals with subjective cognitive decline
Source: Alzheimers Res Ther. 2026 Mar 27;18:104. doi: 10.1186/s13195-026-02026-3 (PMC13151223; doi:10.1186/s13195-026-02026-3)
Supplement: Supplementary file 1 — Supplementary Material 1. [file 13195_2026_2026_MOESM1_ESM.docx]

# **Supplement 2**

Interview guide topic list

**Introduction**

- The interviewer is JS, PhD candidate, research focus on medical-ethical aspects of personalized medicine for Alzheimer’s disease.
- Purpose: to explore long-term experiences of knowing one's amyloid PET result.
- The interview will be audio recorded, transcribed verbatim, and de-identified.
- Transcripts will not be returned for comment or correction.
- Participation is voluntary; withdrawal possible at any time.

**Opening question**

A few years ago, you underwent an amyloid PET scan.

- Why did you want to learn the result?

### **Considerations**

- What considerations played a role in your decision?
- What were your reasons for wanting to know the result?
- Did you also have reasons for not wanting to know?

### **Actionability**

- At the time, were there aspects of your life you intended to change based on the result?
  - Lifestyle, diet, sleep, physical activity, etc.?
  - Will, insurance, advance directives, etc.?
  - Decisions made differently, sooner, or later?

### **Result**

- What was the result of your amyloid PET scan, and what does it imply?
  - Was the scan positive or negative?
  - How do you interpret this result?
  - How certain is the result?

## **Response**

- What was your immediate reaction to learning the result?
  - What emotions did you experience?
- How do you feel about it now?

### **(Un)certainty**

- Has the test result answered your questions?
- Has it also raised new questions?
- Has it provided certainty?
- Has it also provided uncertainty?

### **Awareness**

- What role does the test result have in your life?
  - Do you think about it daily, weekly, monthly?
- When something escapes your mind, how do you feel about that now?
  - Has the test result influenced this?

### **Sharing**

- Have you shared the result of your amyloid PET scan with others?
  - Why or why not?
  - With whom?
  - What do you tell them?
  - How do they respond to this information?
  - Do you have the impression that it affects your relationships?
- Have you told or would you tell your partner, family, friends, neighbors, colleagues, insurer?

### **Behavior**

- Have you made changes in your life based on the result?
  - Administrative matters?
  - Health?
  - Quality of life?
- If you were faced with a choice or opportunity, to what extent does your amyloid PET result influence your decision to do or not do this?
  - For example, taking on a multi-year volunteer role, going on an international trip, or moving to a new home

### **Regret and advice**

- If we could turn back the clock, would you make the same choice again?
- If other people have subjective memory complaints and undergo an amyloid PET scan, and are faced with the choice of whether or not to receive the result, what advice would you give them?

## **Evaluation**

- Do you feel a need for more, less, or different information?

### **Other**

- Are there aspects we have not discussed, but that you think are relevant to mention?
