# Supplementary material for: Long-term impact of disclosing amyloid PET results to individuals with subjective cognitive decline
Source: Alzheimers Res Ther. 2026 Mar 27;18:104. doi: 10.1186/s13195-026-02026-3 (PMC13151223; doi:10.1186/s13195-026-02026-3)
Supplement: Supplementary file 2 — Supplementary Material 2. [file 13195_2026_2026_MOESM2_ESM.docx]

**Illustrative quotes for each theme and subtheme identified through thematic analysis**

| **1. Motivations for learning amyloid PET status** | |
| --- | --- |
| 1.a. Fear of developing AD due to family history of / experience with dementia | “My mother had Alzheimer’s. [...] And it was an absolutely hor-ri-ble process. She changed, became someone completely different. Our sweet, gentle mother became suspicious, angry, and fearful. [...] And I was, I am, very afraid that I will get it too. That is why I want to know whether I have it or not. [...] It is a death sentence. And a cruel journey. I think it is the worst disease there is.” (Participant 4, A-)  “Because by now my mother and my in-laws had all developed dementia and Alzheimer’s, I felt it was important. And you do get a bit scared, like, would I also be at risk genetically...” (Participant 12, A+) |
| 1.b. Gaining insight in current situation / potential future | “I wanted to know what’s going on, and where it’s headed.” (Participant 1, A+)  “I want to know whether I have Alzheimer’s or will develop it.” (Participant 4, A-)  “The main reason was simply that I really wanted to know: what is my condition? How likely am I to develop dementia or Alzheimer’s?” (Participant 5, A-)  “If I know things in advance, I feel more at ease with it than when it happens to me.” (Participant 9, A-)  “I just wanted to know what was going on up there in my brain.” (Participant 17, A+) |
| 1.b.i. Personal values / health heuristics | “You don't just walk away; you'd have to be mad. You wouldn't want them to withhold the results of a cancer test either. Then you're just a fool.” (Participant 1, A+)  “I’m curious and I want to know. The consequences are secondary, I think, because the benefit of knowing is more important to me than anything else.” (Participant 2, A+)  “I'm not the kind of person who buries their head in the sand, like, I don't want to know. If tests are being done, whether the result is negative or A+, I do want to know.” (Participant 8, A-) |
| 1.c. Finding cause of / explanation for subjective complaints | “Well, with all the knowledge about the condition I have... I mean, if you have a headache, you want to know what’s causing it... at least I want to know where it comes from and whether something can be done about it.” (Participant 2, A+)  “At that time, I was very forgetful, when all of that was going on, wondering what was wrong with me. That was during menopause. It affected me deeply. I was acting in theater, and it made me feel insecure, like, I don’t remember things, is something wrong with me?” (Participant 10, A+) |
| 1.d. No drawbacks | “No, none at all. [...] No. I never really stopped to think about it. I was only thinking, I want to know.” (Participant 10, A+)  “A spinal tap or a PET scan doesn’t really bother me. I also don’t see the drawbacks right away.” (Participant 16, A+)  “I hadn’t noticed any progression in the meantime, like not being able to tie my shoelaces or anything like that. So why wouldn’t I want to know if they offer the possibility?” (Participant 18, A+) |
|  | |
| **2. Comprehension of amyloid PET result** | |
| 2.a. Amyloid PET negative: meaning | “I was told that I had a good result. That nothing was found, and that I was fine, healthy. No amyloid detected.” (Participant 4, A-)  “I don’t know anymore, what the result was. I think, if I don’t remember it, then there was nothing special going on. So I think I just stuffed that in my filing cabinet and went on living.” (Participant 7, A-)  “The protein wasn’t detected. And I believe that scan was also based on that: to show whether it’s there or not. And if the protein isn’t there, then, well, Alzheimer’s isn’t there either.” (Participant 8, A-) |
| 2.b. Amyloid PET negative: prognosis | “It wasn’t a 100% guarantee that I’d never get Alzheimer’s. That’s not how it works, of course.” (Participant 6, A-)  “I won’t be affected for the next ten years. Well, for me that’s obviously a huge relief. [...] I was very pleased with the result, of course, but I also don’t need them to give me a guarantee." (Participant 9, A-)  “And it’s not like I feel totally safe for a long time or anything. No, that’s not how I see it. I really saw it as a snapshot, like, okay, for now it looks good.” (Participant 11, A-) |
| 2.c. Amyloid PET positive: meaning | “Well, I mean, they found amyloid. That’s not something you want in your head.” (Participant 1, A+)  “If the amount of amyloid is higher than that limit, then you get notified. So, at some point, I got that message. But you don’t know what the threshold is, you don’t know what it was before, whether it was way above the line, or just barely over. So, basically, you still don’t know much.” (Participant 12, A+)  “[The doctor] told me straight up: ‘It’s bad news. They found amyloid proteins in the back of your brain. […] And yes, that means you basically have the early stage of Alzheimer’s.’” (Participant 13, A+) |
| 2.d. Amyloid PET positive: prognosis | “[It] basically guarantees you’ll almost certainly develop Alzheimer’s.” (Participant 3, A+)  “The doctor did say it’s not set in stone that you’ll develop it. There are other factors that determine that. Things like living healthy, staying active, exercising, you name it. And then they also said that in the first five years, it probably wouldn’t really manifest yet.” (Participant 16, A+)  “It doesn’t get better. It can only get worse. And it can take *years* before it has deteriorated.” (Participant 17, A+) |
|  | |
| **3. Emotional impact of amyloid PET result** | |
| 3.a. Immediate reaction to negative result: happiness and relief | “Thank God, of course I was happy, really happy. Happy, happy, happy” (participant 4, A-)  “I was incredibly happy. Yes, incredibly. Because part of me always keeps in mind that the result could be worse.” (participant 9, A-)  “It’s stressful of course. A test like that can really go either way, and it occupies your mind. […] So, I was very happy with the result. […] It gives you an extra period of peace of mind, like hey, the proteins it could start with aren’t there.” (participant 11, A-) |
| 3.b. Adjusting to a negative result: certainty and acceptance | “I think it gave me a bit of certainty, or took away some doubt, like, couldn’ it be, or isn’t there an increased chance that I’m developing dementia or that I’m developing Alzheimer’s? For me, it just gave me more peace of mind, like, okay, these are the facts for me now, I trust them.” (participant 5, A-)  “It was, of course, a reassuring conclusion, that it’s really not that bad compared to people my age. And maybe even some sort of acceptance, like, well, this is how it is, and we’ll just have to make do with it.” (participant 6, A-) |
| 3.c. Immediate reaction to positive result: shock and dread | “It really hits you, like you’re thinking: whoa, here we go.” (participant 12, A+)  “A huge disappointment. Yes. I thought it was awful to hear. […] He literally said, ‘It’s not good.’ So, yes, well. At any rate, he told me that nothing could be done about it anymore.” (participant 13, A+)  “At first, it really shocked me. How can that be? I feel healthy.” (participant 17, A+)  “The first conversation I had with the neurologist after the PET scan, I found that pretty confronting at the time. I asked a few questions, but beyond that, yeah, I just kind of shut down.” (participant 18, A+) |
| 3.d. Adjusting to a positive result: clarity and resignation | “I’m actually pretty calm about it. I know I’m not going to live forever anyway, and I know that if things stay as they are now and I can go on for a few more years, I’m fine with that.” (participant 1, A+)  “If I didn’t have this knowledge, then you’d be constantly asking yourself: Jesus, why do I keep forgetting all these things all the time? And that’s not exactly pleasant either, of course.” (participant 2, A+)  “Of course, in a way it is a burden, knowing with some certainty that you’re going to get it. But I’ve adjusted my life for that. I wouldn’t have done that otherwise.” (participant 16, A+) |
|  | |
| **4. Personal actionability of amyloid PET status** | |
| 4.a. Optimizing well-being and making the most of time left | “I’ve gained a new awareness of what’s really important and what I truly want. Assuming you only have 15, 10, or 5 conscious years left - though you never know - what do you want then and what don’t you want? So, that awareness has changed somewhat. Priorities have changed too.” (Participant 5, A-)  “I think I say yes more quickly than I used to when people suggest something. Before, I would think, oh, it will happen someday, but now I think, okay, sure. [...] At least you can still do it now, and you don’t know if that will still be possible the next time.” (Participant 14, A+) |
| 4.b. Improving lifestyle / reducing risk / slowing progress | “No, a life you live, or that I live, I’ve been living it for so many years; you don’t make many changes to it anymore. Not drinking anymore would be very good, but I can’t do it.” (Participant 8, A-)  “It serves as a motivator to maintain a good and healthy lifestyle. […] I no longer eat meat. I avoid sugars too. What matters is that my mind stays sharp.” (Participant 10, A+)  “I have, well, what I feel is a healthy diet. I don’t smoke, I don’t drink, and I try to get enough exercise. You know, sure, there’s always something you could do better, but I’m satisfied.” (Participant 9, A-) |
| 4.c. Contribute to or benefit from scientific progress | “I continue to hope for a moment when it can be halted.” (Participant 3, A+)  “If I’m here and I participate, it could also mean that they detect earlier that something is going on. […] Maybe in a few years they’ll say, oh yes, and then you can start medication earlier or something like that. Yes, that would of course be fantastic.” (Participant 11, A-)  “I have been a bit disappointed a few times [...] because I hoped to participate in trials, medication trials. [...] But so far, that has not been possible.” (Participant 16, A+) |
| 4.d. Preparing for or adjusting to cognitive decline | “What I’ve done is make sure all debts are paid. If I pass away, my wife will get a sum of money from the insurance. And, uh, she receives a good pension. […] So, she doesn’t have to worry.” (Participant 3, A+)  “I want to be prepared for the process of Alzheimer’s. [...] Of course, you want to know that for many things, from your life plans to your housing, to, well, your entire social context, actually.” (Participant 6, A-)  “I know there’s nothing I can do to change it. But I believe there is a way to live with it for quite some time. Because you can adjust your life to some extent.” (Participant 7, A-) |
| 4.d.i. Preparing for physician assisted suicide or euthanasia | “I want to know if I need to take measures for myself. And I don’t want to go through the entire course of the disease. I want [...] to stop it early. Life like that has no value for me. And I don’t want my children and my husband to remember me that way.” (Participant 4, A-)  “I believe I have the right to make decisions about my own life.” (Participant 6, A-)  “We also made a living will, which clearly states that if I no longer recognize people, or if I’m far advanced in dementia, then it’s been a good life, and it may come to an end.” (Participant 12, A+) |
|  | |
| **5. Sharing amyloid PET status with others** | |
| 5.a. Openness towards a broad social circle | “With everyone. Because it was positive: I don’t have it. […] Whether that’s smart or not, I don’t know, but that’s just what I do. It’s in my nature to share things I experience with others.” (participant 8, A-)  “I like them to be informed. That’s why they are friends and family – they are in the same category, I think - to keep those people informed, to not hide things that are important for them too.” (participant 14, A+)  “I also have a card club, we play every two weeks, and I told them right away. So, if any symptoms - if I start having trouble remembering the game, well, then they’ll also know the cause.” (participant 16, A+) |
| 5.b. Caution in choosing who to inform | “I could actually talk about it just fine, but especially my wife didn’t want that […] We did have the fear that, well, if your employer finds out, then it’s pretty much the end of your career.” (participant 5, A-)  “I don’t feel like explaining to everyone and their dog what it means to get news like this. I’m fine with having a few people I can talk it over with, that’s good, for me too. But not - no.” (participant 7, A-)  “I really debated whether I should tell my wife. […]. And then I thought, well, in this situation I don’t think I should burden her with that. Let’s just see how things are in a few years.” (participant 18, A+) |
|  |  |
| **6. Perceived significance of amyloid PET testing** | |
| 6.a. Evolving personal significance | “You think more about life being finite. […] Now it’s my turn, and sooner than normal. […] There were things I really should get sorted out someday, now I felt like doing them tomorrow. That’s a bit of an exaggeration, but the clearer you see that period, the more urgency there is.” (participant 2, A+)  “But now we’re years in. And even now I don’t plan on, you know, ending it all right away. I don’t want to die yet. […] I’ve still enjoyed myself, you know? I’ve definitely had a livable life up to now. […] It’s still worth living with my wife in that house. It’s nice, just last night I cooked dinner.” (participant 13, A+) |
| 6.b. No regret, would do it again | “Actually, I would have preferred to do it even earlier. Because for all those years before [you’re always wondering]: do I, don’t I, what’s going on?” (participant 10, A+)  “Even if it’s bad news, I still want to know. Yes, I really do. So in the end I’m glad I found out in time.” (participant 13, A+)  “Trying to live in the present now, and trying to do the things you want to do now, of course you can never have any regrets about [the test and your response] afterwards.” (participant 16, A+) |
| 6.c. Advice to others | “I think everyone should decide that for themselves. I see it as establishing something in a scientific way, but someone else might […] not want to face that reality so directly.” (participant 6, A-)  “I think it really depends on people’s attitudes. If it were up to me, if they asked me for advice, I’d probably first want to hear what their general outlook is on life, and so on.” (participant 11, A-)  “I’d only do it if you have at least three measurements […] if you want to know what the prognosis is.” (participant 18, A+) |
